# phiD12-Like Livestock-Associated Prophages Are Associated With Novel Subpopulations of *Streptococcus agalactiae* Infecting Neonates

**DOI:** 10.3389/fcimb.2019.00166

**Published:** 2019-05-28

**Authors:** Adélaïde Renard, Laurie Barbera, Luka Courtier-Martinez, Sandra Dos Santos, Anne-Sophie Valentin, Laurent Mereghetti, Roland Quentin, Nathalie L. van der Mee-Marquet

**Affiliations:** ^1^Bactéries et Risque Materno-Foetal, UMR 1282, Infectiologie Santé Publique, Université de Tours, Tours, France; ^2^Cellule Régionale d'Epidémiologie Nosocomiale, Centre d'Appui pour la Prévention des Infections Associées aux Soins CPias Centre val de Loire, Service de Bactériologie et Hygiène, Centre Hospitalier Universitaire, Tours, France

**Keywords:** phiD12-like prophage, *Streptococcus agalactiae*, neonates, infection, lysogeny, virulence, adaptation

## Abstract

Group B *Streptococcus* (GBS) is a major cause of invasive disease in neonates worldwide. Monitoring data have revealed a continuing trend toward an increase in neonatal GBS infections, despite the introduction of preventive measures. We investigated this trend, by performing the first ever characterization of the prophage content for 106 GBS strains causing neonatal infections between 2002 and 2018. We determined whether the genome of each strain harbored prophages, and identified the insertion site of each of the prophages identified. We found that 71.7% of the strains carried at least one prophage, and that prophages genetically similar to livestock-associated phiD12, carrying genes potentially involved in GBS pathogenesis (e.g., genes encoding putative virulence factors and factors involved in biofilm formation, bacterial persistence, or adaptation to challenging environments) predominated. The phiD12-like prophages were (1) associated with CC17 and 1 strains (*p* = 0.002), (2) more frequent among strains recovered during the 2011–2018 period than among those from 2002–2010 (*p* < 0.001), and (3) located at two major insertion sites close to bacterial genes involved in host adaptation and colonization. Our data provide evidence for a recent increase in lysogeny in GBS, characterized by the acquisition, within the genome, of genetic features typical of animal-associated mobile genetic elements by GBS strains causing neonatal infection. We suggest that lysogeny and phiD12-like prophage genetic elements may have conferred an advantage on GBS strains for adaptation to or colonization of the maternal vaginal tract, or for pathogenicity, and that these factors are currently playing a key role in the increasing ability of GBS strains to infect neonates.

## Introduction

Bacterial pathogens frequently harbor prophages or phage remnants within their DNA. Prophage-encoded virulence factors have been identified in many bacterial species, including *Staphylococcus aureus* and *Streptococcus pyogenes* (Banks et al., 2002; Brüssow et al., 2004; Denou et al., 2008; Boyd, 2012; Harrison and Brockhurst, 2017). These virulence factors include extracellular toxins, enzymes and proteins altering antigenicity or involved in invasion. Temperate phages may also mediate the adaptation of lysogens to new hosts, thereby increasing their fitness (Brüssow et al., 2004; Chibani-Chennoufi et al., 2004); furthermore, lysogeny contributes to intraspecies genomic diversity in bacteria. Within *Staphylococcus aureus*, the acquisition of particular prophages has been associated with the emergence of lineages adapted to new ecological niches (Lowder et al., 2009; Moon et al., 2015; Diene et al., 2017).

In GBS, lysogeny was first described by Russell in 1969, when temperate phages were isolated from strains of bovine origin (Russell et al., 1969). Whole genome sequencing identified phage-associated genes accounting for 10% of all strain-specific GBS genes (Tettelin et al., 2002). GBS strains from different lineages isolated from infected neonates and adults have been shown to have a greater exposure to lysogeny than colonizing strains (van der Mee-Marquet et al., 2006; Salloum et al., 2011). Recently, the whole-genome sequencing of 14 representative GBS strains led to the definition of six groups of genetically similar prophages: groups A to F. The prophages were found close to bacterial genes, such as the *msrR* and *cop* operons involved in bacterial virulence, and *adaA* and *smr*, associated with host adaptation to environmental stress (Britton et al., 1998; Hubscher et al., 2009; Yang et al., 2011; Singh et al., 2015). An analysis of the prophage content of 275 GBS isolates recovered from colonized and infected humans, revealed frequent lysogeny with prophages of group A (van der Mee-Marquet et al., 2017). Prophages A are genetically similar to the phiD12 prophages recently described in *Streptococcus suis*, which infects livestock (Tang et al., 2013). Prophages A harbor genes that have been shown to protect bacteria against horizontal gene transfer and to mediate adaptation to stress and colonization. They contain, in particular, a gene encoding a hypothetical protein with an LPXTG motif consistent with adhesin function (van der Mee-Marquet et al., 2017), the four genes *clpP, metK, relB*, and *yafQ*, which have been implicated in biofilm formation and resistance to both oxidative and acid stresses (Yadav et al., 2012; Hou et al., 2014; Wen et al., 2014; Chan et al., 2018).

GBS frequently colonizes the gastrointestinal and urogenital tracts of healthy individuals, and is a leading pathogen in neonates (Gibbs et al., 2004; Landwehr-Kenzel and Henneke, 2014). Antibiotic prophylaxis at the time of delivery in colonized parturients has decreased the incidence of neonatal early-onset disease (EOD) (Romain et al., 2018). However, a number of recent epidemiological studies worldwide have shown that GBS continues to be responsible for severe late-onset disease (LOD) in neonates and young children (Joubrel et al., 2015; Nanduri et al., 2019). The mechanisms underlying these trends have yet to be elucidated.

Lysogeny has affected the evolution of numerous bacterial species, modifying fitness and affecting adaptation to new hosts or virulence (Brüssow et al., 2004). We investigated the potential role of particular prophages in the evolution of GBS species, by focusing on lysogeny in GBS strains causing neonatal infections. We studied the prophage content of GBS strains responsible for fetal and neonatal deaths, bloodstream infections and meningitis in French neonates between 2002 and 2018. We determined carriage prevalence for the prophages of each of the six groups, and characterized the insertion sites of the phiD12-like A prophages detected in the strains studied.

## Materials and Methods

### Bacterial Strains

The GBS strains were collected from 106 infected neonates during a three-month annual survey performed every year since 2002 at all the general hospitals and clinics located in the Center-Val de Loire region of France (2.8 million inhabitants). The methods and study design have been reported previously (van der Mee-Marquet et al., 2014). The 106 neonates studied comprised 50 neonates with bloodstream infections, 43 with meningitis and 13 who died during fetal development or in the neonatal period. The major lineages to which the GBS strains belonged were determined by MLST (Jones et al., 2003) (Table 1).

**Table 1:** Prophage content of the 106 GBS strains, determination of prophage A insertion sites.

**Prophage group**	**A**					**B**	**C**	**D**	**E**	**F**
**Prophage A insertion sites**		**IS A1**	**IS A2**	**IS A3**	**IS A4**					
All strains for 2002–2018 (*n* = 106)	46 (43.4)	3	11	7	25	10 (9.4)	13 (12.3)	8 (7.5)	14 (13.2)	17 (16.0)
EOD (*n* = 58)	22 (37.9)	3	6	2	11	2 (3.4)	7 (12.1)	3 (5.1)	8 (13.8)	12 (20.7)
2002–2010 (*n* = 43)	11 (25.6)	1	2	2	6	1 (2.3)	6 (13.9)	2 (4.6)	5 (11.6)	9 (20.9)
2011–2018 (*n* = 15)	11 (73.3)	2	4		5	1 (6.7)	1 (6.7)	1 (6.7)	3 (20.0)	3 (20.0)
LOD (*n* = 48)	24 (50.0)		5	5	14	8 (16.7)	7 (14.6)	5 (10.4)	6 (12.5)	5 (10.4)
2002–2010 (*n* = 34)	13 (38.2)		5	1	7	5 (14.7)	5 (14.7)	4 (11.8)	4 (11.8)	3 (8.8)
2011–2018 (*n* = 14)	11 (78.6)			4	7	3 (21.4)	2 (14.3)	1 (7.1)	2 (14.3)	2 (14.3)
Fetal-neonatal death (*n* = 13)	5 (38.5)		4		1			1 (7.7)	2 (15.4)	2 (15.4)
Bacteremia (*n* = 50)	23 (46.0)	3	2	4	14	7 (14.0)	9 (18.0)	5 (10.0)	6 (12.0)	11 (22.0)
Early (*n* = 31)	15 (48.4)	3	2	2	8	2 (6.5)	5 (16.1)	1 (3.2)	4 (12.9)	6 (19.4)
Late (*n* = 19)	8 (42.1)			2	6	5 (26.3)	4 (21.1)	4 (21.1)	2 (10.5)	5 (26.3)
Meningitis (*n* = 43)	18 (41.9)		5	3	10	2 (4.6)	4 (9.3)	2 (4.6)	6 (14.0)	3 (7.0)
Early (*n* = 14)	2 (14.3)				2		2 (14.3)	1 (7.1)	2 (14.3)	2 (14.3)
2002–2010 (*n* = 13)	1 (7.7)				1		2 (15.4)	1 (7.1)	2 (15.4)	2 (15.4)
2011–2018 (*n* = 1)	1 (100.0)				1		2 (14.3)	1 (7.1)	2 (14.3)	2 (14.3)
Late (*n* = 29)	16 (55.2)		5	3	8	2 (6.9)	2 (6.9)	1 (3.4)	4 (13.8)	1 (3.4)
2002–2010 (*n* = 21)	9 (42.9)		5		4	1 (4.8)	2 (9.5)	1 (4.8)	2 (9.5)	1 (4.8)
2011–2018 (*n* = 8)	7 (87.5)			3	4	1 (12.5)			2 (25.0)	
CC1 (*n* = 10)	8 (80.0)		8							3 (30.0)
CC17 (*n* = 50)	26 (52.0)	1	1	5	19	9 (18.0)	11 (22.0)	5 (10.0)	1 (2.0)	5 (10.0)
2002–2010 (*n* = 34)	13 (38.2)	1	1	1	10	5 (14.7)	9 (26.5)	4 (11.8)		2 (5.9)
2011–2018 (*n* = 16)	13 (81.2)			4	9	4 (25.0)	2 (12.5)	1 (6.2)	1 (6.2)	3 (18.7)
CC19 (*n* = 12)	3 (25.0)		2	1		1 (8.3)	2 (16.7)	2 (16.7)	3 (25.0)	1 (8.3)
CC23 (*n* = 25)	7 (28.0)	1		1	5			1 (4.0)	10 (40.0)	4 (16.0)
Other CCs (*n* = 9)	2 (22.2)		1		1					4 (44.4)

*Number of characterized strains with at least one prophage in their genome (%), by prophage group*.

### Detection of GBS Prophages and Prophage A Insertion Sites

We used a previously published PCR protocol with 14 primer pairs to determine whether the genome of each GBS strain harbored genetic elements from prophages belonging to groups A to F (van der Mee-Marquet et al., 2017). Strains harboring group A prophages were then studied with a multiplex PCR tool, with five primer pairs designed to identify the prophage insertion site. All the primer pairs used are presented on Supplementary Figures. PCR was carried out on a Chromo 4 system instrument (Bio-Rad, Hercules, CA), on DNA isolated from the GBS strains. Amplifications were validated by sequencing one of each amplicon obtained with each formulated primer pair. The detection of an amplicon of the expected size was considered to indicate a positive result.

### Statistics

Fisher's exact tests or chi^2^ tests were used for statistical analysis; *p* values < 0.05 were considered significant.

### Ethics Statement

The neonatal isolates were obtained from clinical samples during annual surveillance studies performed in accordance with French national recommendations for the prevention of infection. This monitoring program was approved by the appropriate national committee: the *Réseau Alerte Investigation Surveillance des Infections Nosocomiales*. In accordance with French legislation, the surveillance study was run jointly by the regional surveillance coordinator, the directors of the participating healthcare institutions and the physicians responsible for patient care. The directors and the physicians provided written consent for participation in the study.

## Results

### Lysogeny and GBS Strains Infecting Neonates (Table 1)

The overall frequency of prophage carriage in the 106 GBS strains studied was 71.7% (76 GBS strains carried at least one prophage). The prophage carriage rate differed between prophage groups. It was highest for group A (46/106, 43.4%) and lowest for groups B and D (9.4 and 8.5%, respectively). The carriage of prophages differed between GBS lineages: prophages A were more frequent in the strains of CC1 and 17 (80 and 52.0%, respectively) than in the strains of other CCs (*p* = 0.002); prophages B were significantly associated with CC17 strains (*p* = 0.004) and prophages E were significantly associated with CC23 strains (*p* = 0.001). The frequency of carriage did not vary with clinical form (fetal or neonatal death, bloodstream infection or meningitis) in any of the prophage groups. However, prophages A were more frequent among strains recovered from cases of late-onset meningitis than among strains causing early-onset meningitis (55.2 vs. 14.3%, *p* = 0.027).

### Changes in Prophage Carriage Rates Between 2002 and 2018 (Table 1)

**Figure 1:**
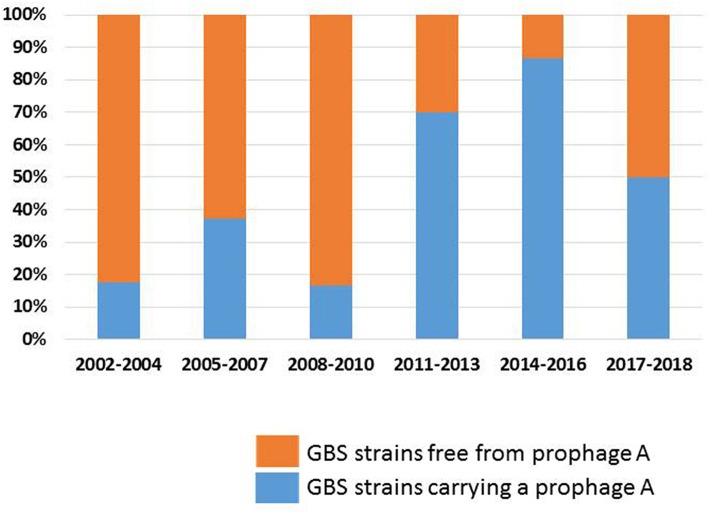
Carriage rate of prophages A among GBS strains; evolution between 2002 and 2018. The proportion of GBS strains carrying a prophage A, and of GBS strains free from prophage A are shown in blue and orange, respectively.

The carriage rates of prophages from groups B, C, D, E, and F did not vary significantly between 2002 and 2018. By contrast, the carriage rate of prophage A increased significantly between 2002 and 2018 (Figure 1): prophages A were more frequent among strains recovered during the 2011–2018 period (75.9%) than among those recovered from 2002 to 2010 (30.8%, *p* < 0.001). The carriage rate of prophages A increased among cases of EOD (73.3 for 2011–2018 vs. 25.6% for 2002–2010, *p* = 0.003) and LOD (78.6 vs. 38.2%, *p* = 0.026), and among CC17 strains (81.2 vs. 38.2%, *p* = 0.011). In addition, the prophage A carriage rate differed between the strains associated with meningitis and fetal or neonatal death: a significant increase was observed between 2002 and 2018 (88.9% for 2011–2018 vs. 29.4% for 2002–2010, *p* = 0.004 for strains causing meningitis; 100% for 2011–2018 vs. 20.0% for 2002–2010, *p* = 0.004 for strains causing fetal or neonatal death).

### Prophages A Insertion Sites (IS)

As prophages A are the commonest prophages, and those associated with the GBS CC17 lineage responsible for most of the invasive infections in neonates, we decided to focus on this prophage group. Four insertion sites have been described for prophages A (van der Mee-Marquet et al., 2017). In the studied strains, the multiple insertion sites for prophages A were not used concomitantly, and most of the prophages A were located at IS A4 (54.3%) and IS A2 (23.9%). The location of the prophages did not differ between the clinical forms, or during the study period. IS A4, located close to host genes involved in adaptation and colonization, such as *adaA* and the *murB*-*potABCD* operon (Samartzidou et al., 2003; Ware et al., 2006), was the predominant insertion site for the prophages A carried by GBS of CC17, and was significantly more frequently occupied by prophages A in strains of CC17 and 23 than in strains from the other CCs (72.7% for CC17 and 23 vs. 7.7% for the other CCs, *p* < 0.001). IS A2 was frequently co-occupied by a prophage and one or two transposons. It is close to host genes, such as *rlmCD*, implicated in DNA repair and *msrR*, which have been shown to increase virulence in *S. aureus* (Hubscher et al., 2009; van der Mee-Marquet et al., 2017). IS A2 was the predominant insertion site for prophages A in CC1 strains, and was significantly more frequently occupied by prophages A in strains of CC1 and 19 than in strains from the other CCs (90.9% for CC1 and 19 vs. 5.7% for other CCs, *p* < 0.001).

## Discussion

We report here the first characterization of the prophages carried by a collection of GBS isolates causing neonatal infections over the two last decades in the Center region of France, providing insight into the ongoing evolution of GBS.

First, we showed that lysogeny is frequently observed in GBS strains responsible for neonatal infections (71.7%), mostly associated with prophages A, which are genetically similar to the phiD12 prophages recently described in livestock-associated *S. suis* (Tang et al., 2013). Second, we showed that the prophage A carriage rate sharply increased during the 2002–2018 period, particularly in the strains of CC17, the clonal complex currently responsible for the largest proportion of early- and late-onset neonatal GBS infections.

Temperate phages affect bacteria by protecting against lytic infection and by lysing competing strains through prophage induction (Brüssow et al., 2004). As suggested by experiments with cocultures of *Salmonella* strains carrying or lacking specific prophages, prophages may modify the competitive fitness of host strains through their ability to grow and, for a small proportion of the population, to annihilate rival bacteria through cell lysis. Phages active against isolates other than the strain causing disease were released from most of the bacterial isolates obtained patients with septicemia, suggesting that the prophages present in sepsis-causing bacterial clones play a role in clonal selection during bacterial invasion (Bossi et al., 2003). Phages originating from the genetically homogeneous CC17 clonal complex have been shown to have a broad spectrum of lytic activities against strains of most GBS lineages (Domelier et al., 2009). Given the particular ability of the phages of CC17 strains to destroy extraclonal strains, such phenomena could confer a selective advantage for vaginal colonization. Chiefly, phiD12-like A prophages carry genes potentially involved in GBS pathogenesis, such as a gene encoding a hypothetical protein possessing an LPXTG motif consistent with adhesion function, and the four genes *clpP, metK, relB*, and *yafQ*, which have been implicated in biofilm formation and resistance to both oxidative and acid stresses (Yadav et al., 2012; Hou et al., 2014; Wen et al., 2014; Chan et al., 2018). Taking into account the ability of rival subpopulations of GBS strains to compete with each other, the production of bacterial adhesins, and the ability to form biofilms, all of which are important characteristics for bacterial pathogenesis and persistent colonization, we hypothesize that lysogeny with phiD12-like A prophages increases the ability of the CC17 strains to colonize the acidic environment in the vagina, thereby favoring maternofetal infection. Further studies are required to test this hypothesis. We have recently constructed several mutants from a lysogenic CC17 GBS strain, one in which the whole prophage has been deleted and others in which different prophagic genes have been deleted. We are currently comparing the properties of the lysogen and mutants, to investigate the functional impact of phiD12-like A prophages on biofilm formation and ability to deal with acid stress.

Temperate phages may affect bacterial fitness by modifying anchor points for genome rearrangements or by disrupting genes (Brüssow et al., 2004). We report the first characterization of the insertion sites of each phiD12-like A prophage identified in the GBS isolates causing neonatal infections. The insertion sites differed significantly between GBS lineages: IS A4 was the predominant insertion site for prophages A in the genome of CC17 strains, whereas, IS A2 predominated in CC1 strains. As noted above, several bacterial genes potentially involved in host resistance to stress conditions, adaptation and colonization have been identified close to theses insertion sites. We investigated the possible co-transcription of prophage and bacterial genes, by performing an *in silico* analysis with BPROM software, to detect promoters in the prophage ORFs directly upstream from the bacterial ORFs, or in the bacterial ORFs upstream from the prophage ORFs (Solovyev and Salamov, 2011). This analysis revealed short intergenic regions (38–249 bp long) between the last prophage ORF (encoding a putative Tn3 transposon revolvase for IS A1, IS A3 and IS A4, and *pknB* for IS A2) and the first bacterial ORF downstream from the prophage (encoding a putative phosphatase (IS A1), *ybjl* (IS A3 and IS A4), or a hypothetical protein (IS A2), respectively). Given that prophage and bacterial ORFs were transcribed in the same direction, and that promoters were detected upstream from the translation initiation site of the prophage ORF and within the prophage ORF at a distance ranging from 179 to 1,481 bp from the first codon of the bacterial ORF, we suggest possible co-transcription of the prophage and bacterial ORFs under the control of prophage promoters. By contrast, the analysis provided no evidence for the probable co-transcription of the bacterial ORFs upstream from the prophage and the first prophage ORFs. Indeed, the distance separating bacterial and prophage ORFs was too large (IS A1 and IS A3), or the ORFs were not transcribed in the same direction (IS A4). Only for IS A2, for which there was a short distance between the last ORF (encoding *mepA*) of the transposon and the first prophage ORF (encoding an RNA polymerase), and promoters located upstream from the transcription initiation site of *mepA* and within the prophage ORF, would co-transcription of the prophage and bacterial ORFs be possible, under the control of *mepA* promoters. Based on bioinformatics analyses, these findings remain speculative and require confirmation in further experiments.

Additional investigations should be performed to explore the impact of prophage insertion site on GBS pathogenicity in neonatal infections. Our data suggest that phiD12-like A prophages, located at different insertion sites, belong to lineage-specific pathogenicity island-like elements on the bacterial chromosome, and that they confer an advantage on GBS strains for adaptation to or colonization of the maternal vaginal tract (Boyd et al., 2000; Boyd, 2012; Almeida et al., 2017).

## Conclusion

The monitoring of neonatal GBS infections in various countries has recently demonstrated a continuing trend toward late-onset neonatal GBS infections, despite the introduction of preventive measures (Joubrel et al., 2015; Nanduri et al., 2019). Our molecular data provide evidence for the recent emergence of novel subpopulations of CC17 strains responsible for neonatal meningitis, and CC1 strains causing fetal or neonatal death, following the acquisition, by their genomes, of genetic features typical of animal-associated MGEs, e.g., phiD12-like A prophages. We suggest that these genetic events may have played a role in the epidemiological changes currently observed for GBS species, including the trend toward an increase in LOD.

Lastly, in account of recent epidemiologic changes with GBS infections in adults, our findings encourage studying the evolution of the lysogeny in GBS strains of CC1, the major clonal complex involved with severe cutaneous, and osteo-articular infections that are increasingly described worldwide in adults (Björnsdóttir et al., 2016).

## Author Contributions

NvdM-M designed the study. AR, LB, LC-M, and SD performed the technical part. AR, A-SV, LM, RQ, and NvdM-M discussed the results. AR and NvdM-M wrote the paper.

### Conflict of Interest Statement

The authors declare that the research was conducted in the absence of any commercial or financial relationships that could be construed as a potential conflict of interest.

## Supplementary Material

Figure S1 Primers used for detection of prophages (A–F) into the bacterial genome, and for the determination of prophage A insertion sites.

## References

[B1] Almeida A. Rosinski-Chupin I. Plainvert C. Douarre P. E. Borrego M. J. Poyart C.. (2017). Parallel evolution of group B *Streptococcus* hypervirulent clonal complex 17 unveils new pathoadaptive mutations. mSystems 2:17. 10.1128/mSystems.00074-17 28904998 PMC5585690

[B2] Banks D. J. Beres S. B. Musser J. M. (2002). The fundamental contribution of phages to GAS evolution, genome diversification and strain emergence. Trends Microbiol. 10, 515–521. 10.1016/s0966-842x(02)02461-7 12419616

[B3] Björnsdóttir E. S. Martins E. R. Erlendsdóttir H. Haraldsson G. Melo-Cristino J. Kristinsson K. G.. (2016). Changing epidemiology of group B streptococcal infections among adults in Iceland: 1975–2014. Clin. Microbiol. Infect. 22, 379.e9–379.e316. 10.1016/j.cmi.2015.11.020 26691681

[B4] Bossi L. Fuentes J. A. Mora G. Figueroa-Bossi N. (2003). Prophage contribution to bacterial population dynamics. J. Bacteriol. 185, 6467–6471. 10.1128/jb.185.21.6467-6471.2003 14563883 PMC219396

[B5] Boyd E. F. (2012). Bacteriophage-encoded bacterial virulence factors and phage-pathogenicity island interactions. Adv. Virus Res. 82, 91–118. 10.1016/B978-0-12-394621-8.00014-5 22420852

[B6] Boyd E. F. Moyer K. E. Shi L. Waldor M. K. (2000). Infectious CTXPhi and the vibrio pathogenicity island prophage in *Vibrio mimicus*: evidence for recent horizontal transfer between *V. mimicus* and *V. cholerae*. Infect. Immun. 68, 1507–1513. 10.1128/iai.68.3.1507-1513.2000 10678967 PMC97308

[B7] Britton R. A. Lin D. C. Grossman A. D. (1998). Characterization of a prokaryotic SMC protein involved in chromosome partitioning. Genes Dev. 12, 1254–1259. 9573042 10.1101/gad.12.9.1254 PMC316777

[B8] Brüssow H. Canchaya C. Hardt W. D. (2004). Phages and the evolution of bacterial pathogens: from genomic rearrangements to lysogenic conversion. Microbiol. Mol. Biol. Rev. 68, 560-602. 10.1128/MMBR.68.3.560-602.2004 15353570 PMC515249

[B9] Chan W. T. Domenech M. Moreno-Cordoba I. Navarro-Martinez V. Nieto C. Moscoso M. (2018). The *Streptococcus pneumoniae* yefM-yoeB and relBE toxin-antitoxin operons participate in oxidative stress and biofilm formation. Toxins 10:378 10.3390/toxins10090378 PMC6162744 30231554

[B10] Chibani-Chennoufi S. Bruttin A. Dillmann M. L. Brüssow H. (2004). Phage-host interaction: an ecological perspective. J. Bacteriol. 186, 3677–3686. 10.1128/JB.186.12.3677-3686.2004 15175280 PMC419959

[B11] Denou E. Pridmore R. D. Ventura M. Pittet A. C. Zwahlen M. C. Berger B.. (2008). The role of prophage for genome diversification within a clonal lineage of *Lactobacillus johnsonii*: characterization of the defective prophage LJ771. J. Bacteriol. 190, 5806–5813. 10.1128/JB.01802-07 18515417 PMC2519514

[B12] Diene S. M. Corvaglia A. R. François P. van der Mee-Marquet N. Regional Infection Control Group of the Centre R. (2017). Prophages and adaptation of *Staphylococcus aureus* ST398 to the human clinic. BMC Genomics 18:133. 10.1186/s12864-017-3516-x 28166723 PMC5294865

[B13] Domelier A. S. van der Mee-Marquet N. Sizaret P. Y. Héry-Arnaud G. Lartigue M. F. Mereghetti L.. (2009). Molecular characterization and lytic activities of *Streptococcus agalactiae* bacteriophages and determination of lysogenic-strain features. J. Bacteriol. 191, 4776–4785. 10.1128/JB.00426-09 19465660 PMC2715722

[B14] Gibbs R. S. Schrag S. Schuchat A. (2004). Perinatal infections due to group B streptococci. Obstet. Gynecol. 104 (5 Pt 1), 1062–1076. 10.1097/01.AOG.0000144128.03913.c2 15516403

[B15] Harrison E. Brockhurst M. A. (2017). Ecological and evolutionary benefits of temperate phage: what does or doesn't kill you makes you stronger. Bioessays 39:112 10.1002/bies.201700112 28983932

[B16] Hou X. H. Zhang J. Q. Song X. Y. Ma X. B. Zhang S. Y. (2014). Contribution of ClpP to stress tolerance and virulence properties of *Streptococcus mutans*. J. Basic Microbiol. 54, 1222–1232. 10.1002/jobm.201300747 24979467

[B17] Hübscher J. McCallum N. Sifri C. D. Majcherczyk P. A. Entenza J. M. Heusser R.. (2009). MsrR contributes to cell surface characteristics and virulence in *Staphylococcus aureus*. FEMS Microbiol. Lett. 295, 251–260. 10.1111/j.1574-6968.2009.01603.x 19459977

[B18] Jones N. Bohnsack J. F. Takahashi S. Oliver K. A. Chan M. S. Kunst F.. (2003). Multilocus sequence typing system for group B streptococcus. J. Clin. Microbiol. 41, 2530–2536. 10.1128/jcm.41.6.2530-2536.2003 12791877 PMC156480

[B19] Joubrel C. Tazi A. Six A. Dmytruk N. Touak G. Bidet P.. (2015). Group B *Streptococcus neonatal* invasive infections, France 2007–2012. Clin. Microbiol. Infect. 21, 910–916. 10.1016/j.cmi.2015.05.039 26055414

[B20] Landwehr-Kenzel S. Henneke P. (2014). Interaction of *Streptococcus agalactiae* and cellular innate immunity in colonization and disease. Front. Immunol. 5:519. 10.3389/fimmu.2014.00519 25400631 PMC4212683

[B21] Lowder B. V. Guinane C. M. Ben Zakour N. L. Weinert L. A. Conway-Morris A. Cartwright R. A.. (2009). Recent human-to-poultry host jump, adaptation, and pandemic spread of *Staphylococcus aureus*. Proc. Natl. Acad. Sci. U.S.A. 106, 19545–19550. 10.1073/pnas.0909285106 19884497 PMC2780746

[B22] Moon B. Y. Park J. Y. Hwang S. Y. Robinson D. A. Thomas J. C. Fitzgerald J. R.. (2015). Phage-mediated horizontal transfer of a *Staphylococcus aureus* virulence-associated genomic island. Sci. Rep. 5:9784. 10.1038/srep09784 25891795 PMC4402969

[B23] Nanduri S. A. Petit S. Smelser C. Apostol M. Alden N. B. Harrison L. H. (2019). Epidemiology of invasive early-onset and late-onset group B streptococcal disease in the United States, 2006 to 2015: multistate laboratory and population-based surveillance. JAMA Pediatr. 173:224 10.1001/jamapediatrics.2018.4826 PMC6439883 30640366

[B24] Romain A. S. Cohen R. Plainvert C. Joubrel C. Béchet S. Perret A.. (2018). Clinical and laboratory features of group B *Streptococcus meningitis* in infants and newborns: study of 848 cases in France, 2001–2014. Clin. Infect. Dis. 66, 857–864. 10.1093/cid/cix896 29045606

[B25] Russell H. Norcross N. L. Kahn D. E. (1969). Isolation and characterization of Streptococcus agalactiae bacteriophage. J. Gen. Virol. 5, 315–317. 10.1099/0022-1317-5-2-315 5347408

[B26] Salloum M. van der Mee-Marquet N. Valentin-Domelier A. S. Quentin R. (2011). Diversity of prophage DNA regions of *Streptococcus agalactiae* clonal lineages from adults and neonates with invasive infectious disease. PLoS ONE 6:e20256. 10.1371/journal.pone.0020256 21633509 PMC3102099

[B27] Samartzidou H. Mehrazin M. Xu Z. Benedik M. J. Delcour A. H. (2003). Cadaverine inhibition of porin plays a role in cell survival at acidic pH. J. Bacteriol. 185, 13–19. 10.1128/jb.185.1.13-19.2003 12486035 PMC141942

[B28] Singh K. Senadheera D. B. Lévesque C. M. Cvitkovitch D. G. (2015). The copYAZ Operon Functions in Copper Efflux, Biofilm Formation, Genetic Transformation, and Stress Tolerance in *Streptococcus mutans*. J. Bacteriol. 197, 2545–2557. 10.1128/JB.02433-14 26013484 PMC4518833

[B29] Solovyev V. Salamov A. (2011). Automatic annotation of microbial genomes and metagenomic sequences, in Metagenomics and Its Applications in Agriculture, Biomedicine and Environmental Studies, ed Li R. W. (Hauppauge, NY: Nova Science Publishers), 61–78.

[B30] Tang F. Bossers A. Harders F. Lu C. Smith H. (2013). Comparative genomic analysis of twelve *Streptococcus suis* (pro)phages. Genomics 101, 336–344. 10.1016/j.ygeno.2013.04.005 23587535

[B31] Tettelin H. Masignani V. Cieslewicz M. J. Eisen J. A. Peterson S. Wessels M. R.. (2002). Complete genome sequence and comparative genomic analysis of an emerging human pathogen, serotype V *Streptococcus agalactiae*. Proc. Natl. Acad. Sci. U.S.A. 99, 12391–12396. 10.1073/pnas.182380799 12200547 PMC129455

[B32] van der Mee-Marquet N. Diene S. M. Barbera L. Courtier-Martinez L. Lafont L. Ouachée A.. (2017). Analysis of the prophages carried by human infecting isolates provides new insight into the evolution of Group B *Streptococcus* species. Clin. Microbiol. Infect. 24, 514–521. 10.1016/j.cmi.2017.08.024 28870726

[B33] van der Mee-Marquet N. Domelier A. S. Mereghetti L. Lanotte P. Rosenau A. van Leeuwen W.. (2006). Prophagic DNA fragments in *Streptococcus agalactiae* strains and association with neonatal meningitis. J. Clin. Microbiol. 44, 1049–1058. 10.1128/JCM.44.3.1049-1058.2006 16517893 PMC1393083

[B34] van der Mee-Marquet N. L. Corvaglia A. Haenni M. Bertrand X. Franck J. B. Kluytmans J.. (2014). Emergence of a novel subpopulation of CC398 *Staphylococcus aureus* infecting animals is a serious hazard for humans. Front. Microbiol. 5:652. 10.3389/fmicb.2014.00652 25538688 PMC4257084

[B35] Ware D. Jiang Y. Lin W. Swiatlo E. (2006). Involvement of potD in *Streptococcus pneumoniae* polyamine transport and pathogenesis. Infect. Immun. 74, 352–361. 10.1128/IAI.74.1.352-361.2006 16368990 PMC1346612

[B36] Wen Y. Behiels E. Devreese B. (2014). Toxin-Antitoxin systems: their role in persistence, biofilm formation, and pathogenicity. Pathog. Dis. 70, 240–249. 10.1111/2049-632X.12145 24478112

[B37] Yadav M. K. Chae S. W. Song J. J. (2012). Effect of 5-azacytidine on *in vitro* biofilm formation of *Streptococcus pneumoniae*. Microb. Pathog. 53, 219–226. 10.1016/j.micpath.2012.08.003 22963864

[B38] Yang M. Aamodt R. M. Dalhus B. Balasingham S. Helle I. Andersen P.. (2011). The ada operon of *Mycobacterium tuberculosis* encodes two DNA methyltransferases for inducible repair of DNA alkylation damage. DNA Repair 10, 595–602. 10.1016/j.dnarep.2011.03.007 21570366

