# Supplementary material for: phiD12-Like Livestock-Associated Prophages Are Associated With Novel Subpopulations of Streptococcus agalactiae Infecting Neonates
Source: Front Cell Infect Microbiol. 2019 May 28;9:166. doi: 10.3389/fcimb.2019.00166 (PMC6546898; doi:10.3389/fcimb.2019.00166)
Supplement: Figure S1 — Primers used for detection of prophages (A–F) into the bacterial genome, and for the determination of prophage A insertion sites. [file Presentation_1.PPTX]

## Slide 1
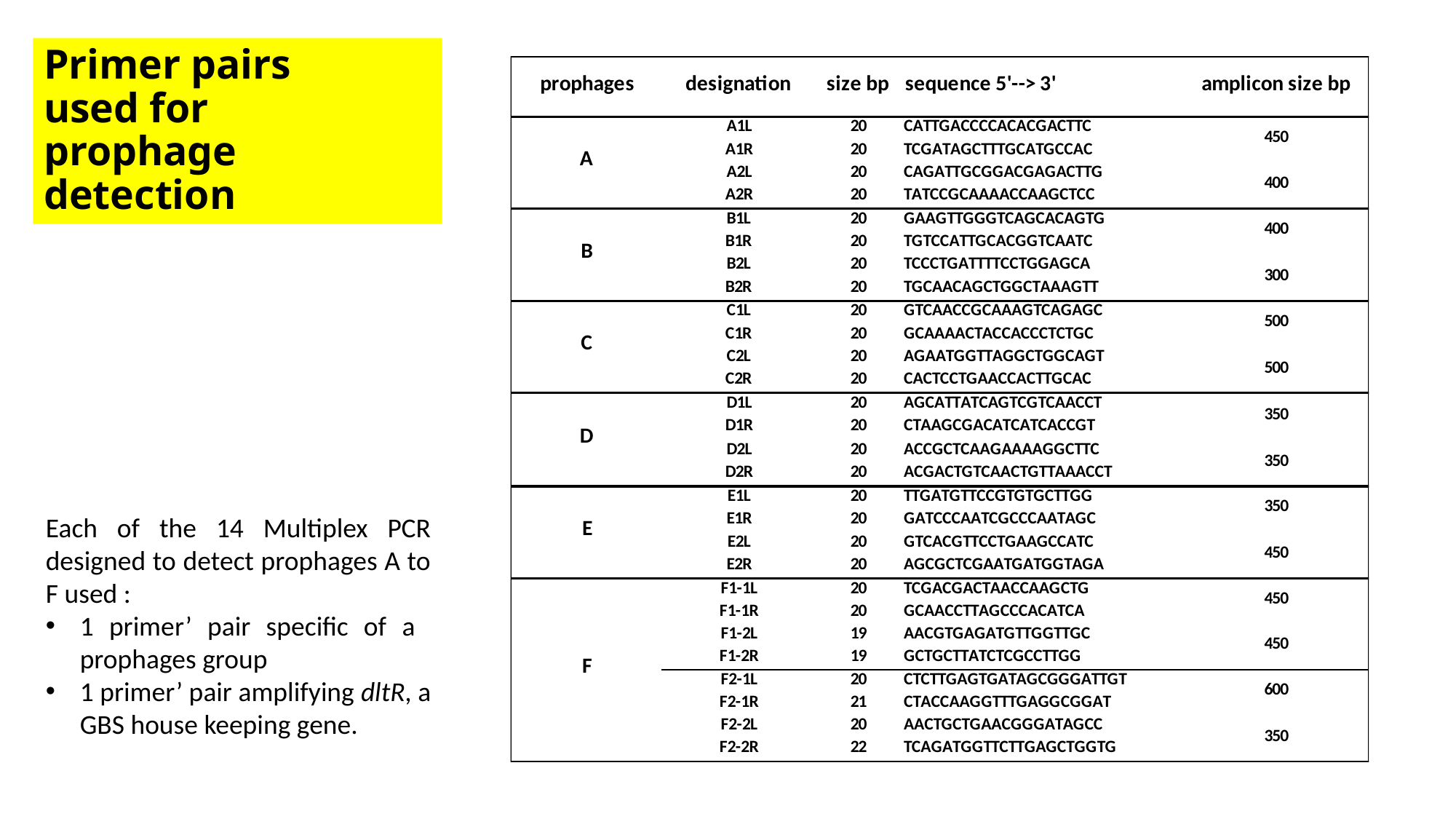

# Primer pairs used for prophage detection
Each of the 14 Multiplex PCR designed to detect prophages A to F used :
1 primer’ pair specific of a prophages group
1 primer’ pair amplifying dltR, a GBS house keeping gene.

## Slide 2
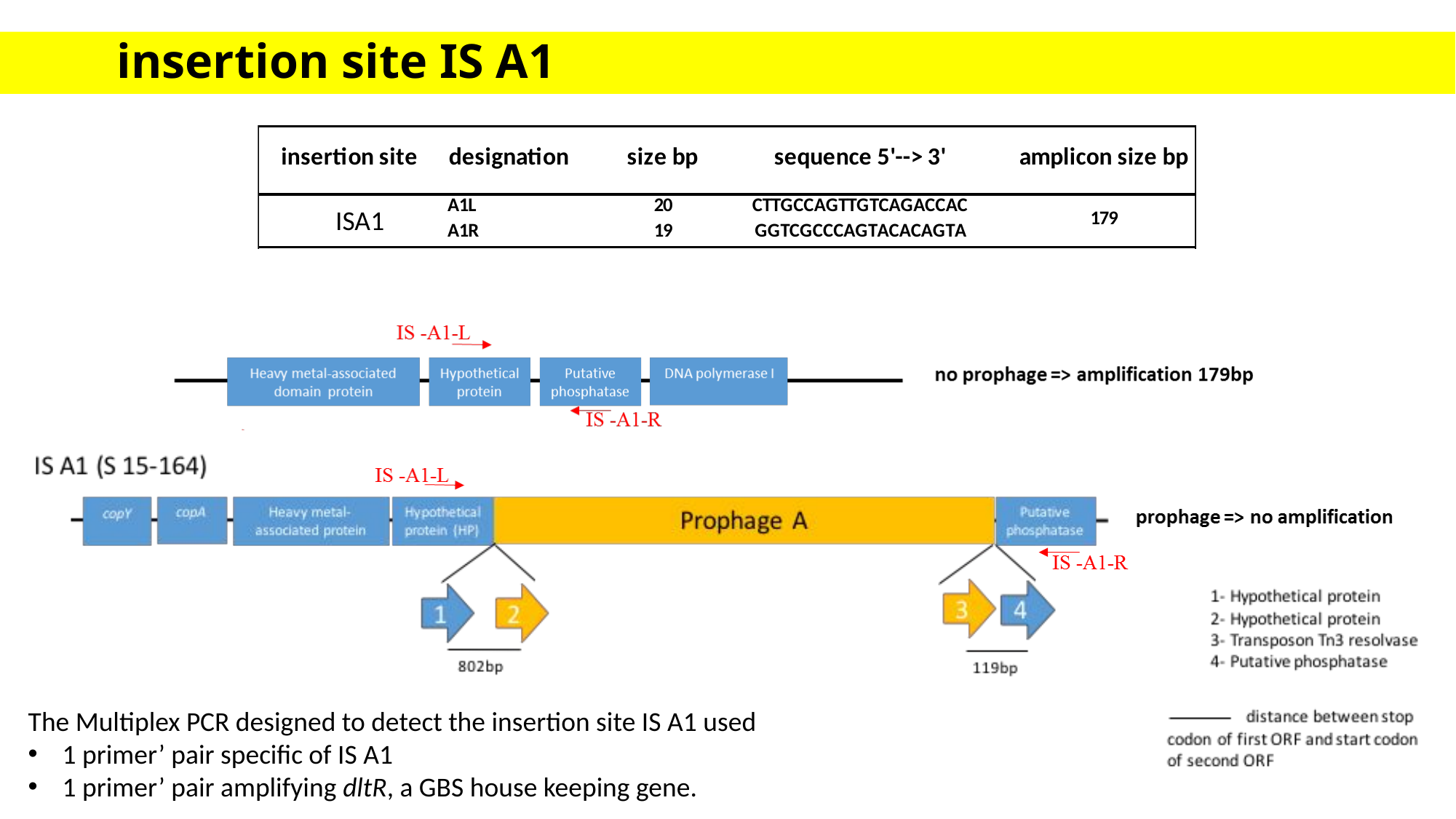

insertion site IS A1
ISA1
The Multiplex PCR designed to detect the insertion site IS A1 used
1 primer’ pair specific of IS A1
1 primer’ pair amplifying dltR, a GBS house keeping gene.

## Slide 3
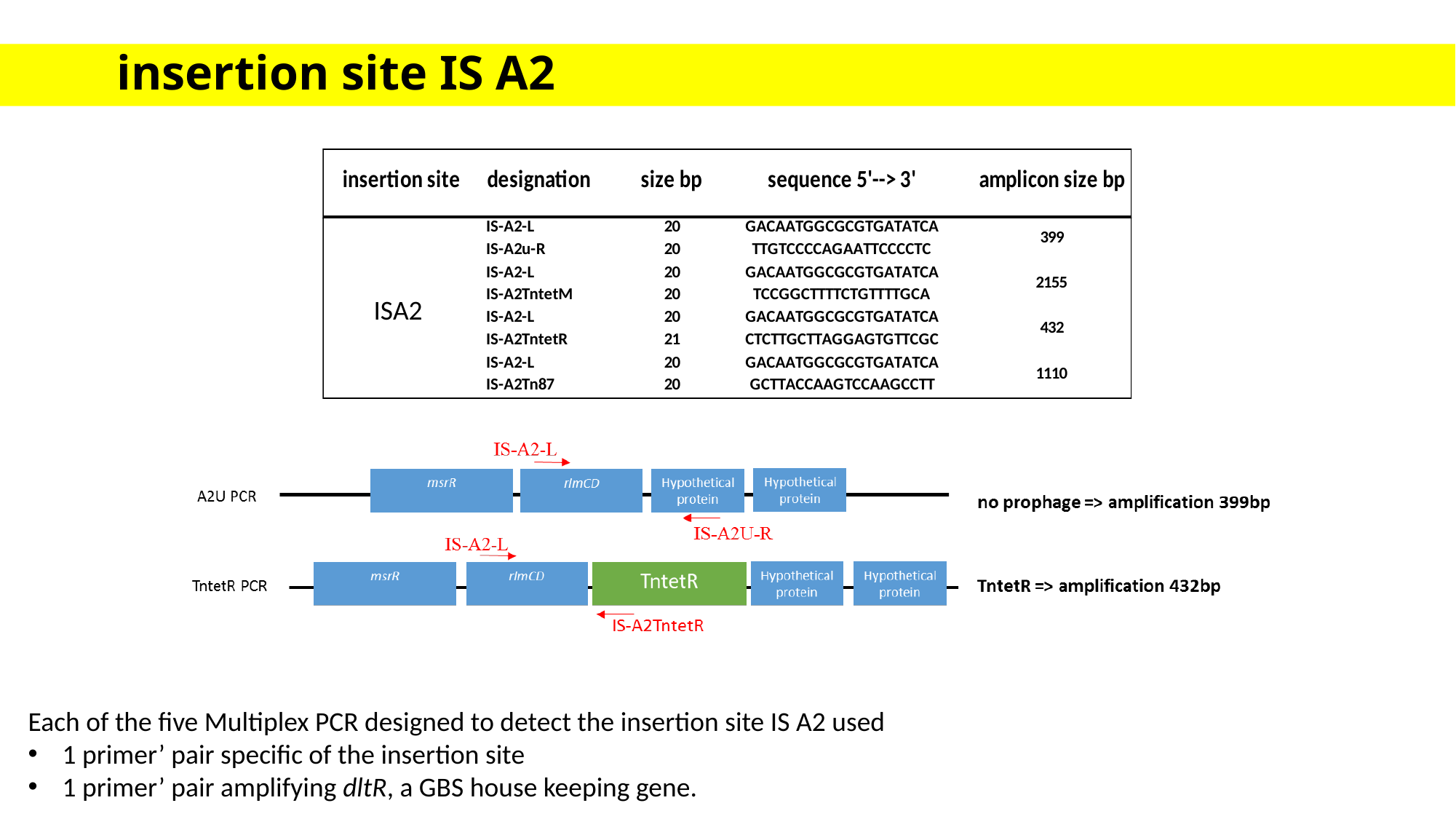

insertion site IS A2
ISA2
Each of the five Multiplex PCR designed to detect the insertion site IS A2 used
1 primer’ pair specific of the insertion site
1 primer’ pair amplifying dltR, a GBS house keeping gene.

## Slide 4
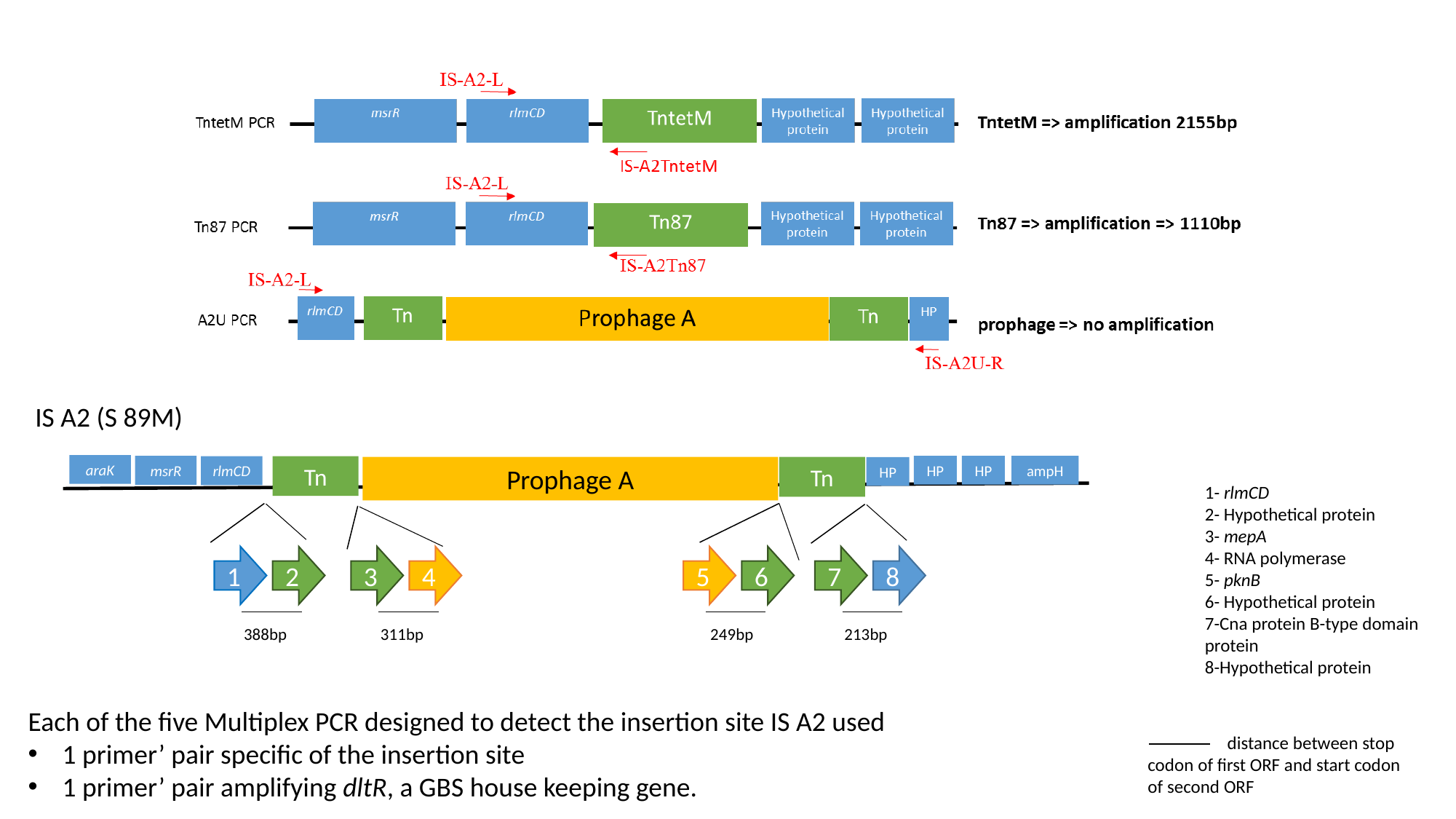

IS A2 (S 89M)
araK
HP
HP
rlmCD
HP
Tn
Tn
Prophage A
1
2
3
4
5
6
7
8
388bp
311bp
249bp
213bp
ampH
msrR
1- rlmCD
2- Hypothetical protein
3- mepA
4- RNA polymerase
5- pknB
6- Hypothetical protein
7-Cna protein B-type domain protein
8-Hypothetical protein
Each of the five Multiplex PCR designed to detect the insertion site IS A2 used
1 primer’ pair specific of the insertion site
1 primer’ pair amplifying dltR, a GBS house keeping gene.
 distance between stop codon of first ORF and start codon of second ORF

## Slide 5
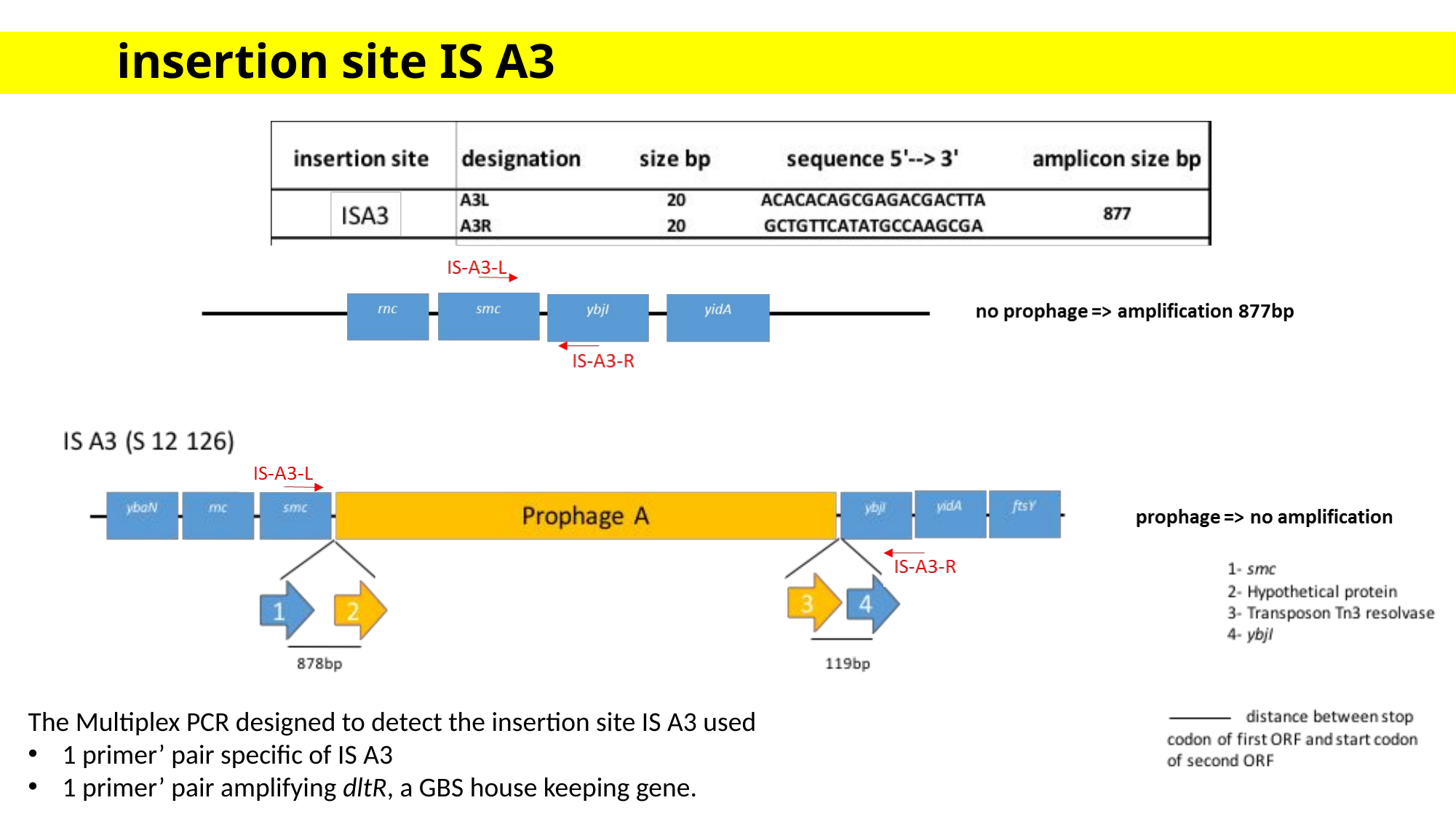

insertion site IS A3
The Multiplex PCR designed to detect the insertion site IS A3 used
1 primer’ pair specific of IS A3
1 primer’ pair amplifying dltR, a GBS house keeping gene.

## Slide 6
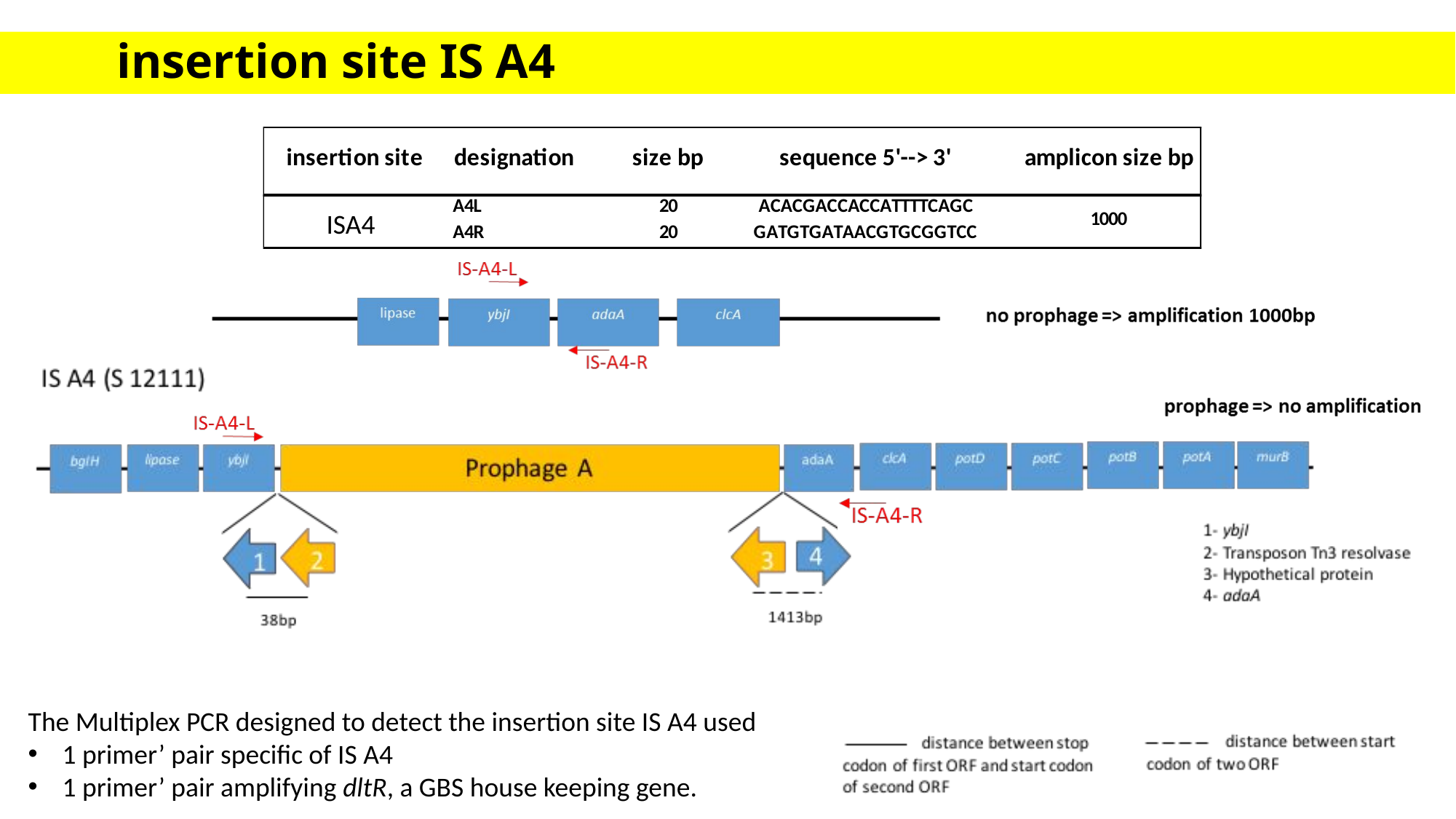

insertion site IS A4
ISA4
The Multiplex PCR designed to detect the insertion site IS A4 used
1 primer’ pair specific of IS A4
1 primer’ pair amplifying dltR, a GBS house keeping gene.
